# A comparison of NAFLD and MAFLD diagnostic criteria in contemporary urban healthy adults in China: a cross-sectional study

**DOI:** 10.1186/s12876-022-02576-4

**Published:** 2022-11-19

**Authors:** Qiling Liu, Gang Zhao, Qian Li, Weiyun Wu, Yan Zhang, Hua Bian

**Affiliations:** 1 Department of Endocrinology and Metabolism, Zhongshan Hospital, Fudan University, Shanghai, China; 2 Fudan Institute for Metabolic Disease, Fudan University, Shanghai, China; 3 Health Examination Center, Wusong Branch of Zhongshan Hospital, Fudan University, Shanghai, China; 4 Clinical Laboratory, Wusong Branch of Zhongshan Hospital, Fudan University, Shanghai, China; 5 Clinical Laboratory, Zhongshan Hospital, Fudan University, Shanghai, China; 6 Department of Endocrinology and Metabolism, Wusong Branch of Zhongshan Hospital, Fudan University, Shanghai, China

**Keywords:** Fatty liver, Metabolic dysfunction-associated fatty liver disease, Nonalcoholic fatty liver disease, Diagnosis

## Abstract

**Background:**

A recently proposed diagnostic criteria of metabolic dysfunction-associated fatty liver disease (MAFLD) is more available for various clinical situations than nonalcoholic fatty liver disease (NAFLD), but understanding about differences between NAFLD and MAFLD in clinical practice remains limited in the general adult urban population in China.

**Methods:**

A total of 795 subjects were recruited from Wu Song Branch of Zhongshan Hospital who participated in the general health assessment. Examination results was obtained through analysis of blood samples and abdominal ultrasonography. Participants were divided into four subgroups according to whether they had NAFLD or MAFLD (NAFLD- MAFLD-, NAFLD + MAFLD-, NAFLD- MAFLD + and NAFLD + MAFLD+).

**Results:**

Among the urban healthy adults investigated, 345 people (43.4%) were diagnosed with NAFLD and 356 people (44.8%) with MAFLD. No significant differences in the prevalence, age, fasting blood glucose, glycosylated hemoglobin, liver enzyme examination, percentage of overweight, hypertension or dyslipidaemia were found between NAFLD and MAFLD patients. Patients with MAFLD had worse metabolic disorders than NAFLD + MAFLD- patients. The NAFLD fibrosis score (NFS) of the NAFLD- MAFLD + group was higher than that of the NAFLD + MAFLD- group. Higher proportion of patients in the NAFLD- MAFLD + group have NFS ≥-1.455.

**Conclusion:**

MAFLD criteria have similar prevalence and patient characteristics compared with previous NAFLD but help to identify a group of patients with high risks of metabolic disorders and liver fibrosis who have been missed with NAFLD, and has superior utility.

## Background

At the beginning of 2020, an international expert group proposed the definition of MAFLD. As a more positive inclusion criterion, which is no longer an exclusive diagnosis like NAFLD, MAFLD diagnostic criteria can identify a more comprehensive cluster of patients with hepatic steatosis who have high risks of metabolic disorders[1, 2]. As a group of acquired metabolic stress-related liver diseases with a prevalence of approximately 38.77%, MAFLD is the most prevalent liver disease at present[3, 4].

NAFLD diagnostic criteria were widely used in clinical practice before MAFLD. NAFLD refers to excessive fat deposition in hepatocytes, which is diagnosed after excluding excessive drinking and other clear factors leading to liver injury[5]. However, with the increase in the prevalence of liver steatosis caused by various factors, NAFLD diagnostic criteria gradually become difficult to meet the needs of clinical work. As an exclusion criterion, the diagnosis of NAFLD needs to exclude liver diseases caused by excessive drinking, drug-induced liver steatosis, autoimmune hepatitis, chronic viral hepatitis, some types of systemic diseases or other reasons that may cause liver injury. As the prevalence increases in the population, the complexity of its clinical application makes NAFLD patient management difficult. NAFLD criteria is not practical, and ignores a subset of patients who have NAFLD characteristics combined with other chronic liver diseases[6]. NAFLD makes it difficult to clarify the connotation of liver steatosis and the actual situation of patients with fatty liver, which reduces people’s vigilance regarding the heterogeneity within NAFLD patients.

Previous studies have shown that NAFLD may aggravate or induce insulin resistance and affect blood glucose management in patients with type 2 diabetes mellitus (T2DM). NAFLD can also predict the occurrence of T2DM as well as cardiovascular disease (CVD) [7, 8]. The close relationship among fatty liver and diabetes, hypertension, and proteinuria has gradually attracted attention, but a convenient and independent diagnostic standard is still needed for general clinical conditions[9–12]. In comparison with patients with T2DM without NAFLD, patients who have T2DM complicated with NAFLD exhibit increased insulin resistance, glucose metabolism disorder, lipid metabolism disorder and serum liver enzyme levels. A close relationship is noted between NAFLD and metabolic disorders, which suggests that NAFLD is not a simple lesion of the liver itself, but a multiorgan disease. NAFLD is a pathological phenomenon observed after multisystem metabolic disorder affects the liver, and this notion is difficult to simply summarize using the current definition of NAFLD[13, 14]. The new MAFLD definition is based on metabolic disorder, which can match the clinical situation of patients with fatty liver more accurately than NAFLD diagnostic criteria[15, 16]. As an inclusive definition, the diagnosis of MAFLD can coexist with other diseases that lead to liver steatosis. In the clinic, MAFLD can be regarded as an independent systemic disease, and its diagnosis is directly based on metabolic abnormalities together with liver findings, efficiently facilitating the classification of patients with liver steatosis confirmed by various methods[17].

Some NAFLD patients will progress to nonalcoholic steatohepatitis (NASH), hepatic fibrosis and hepatocellular carcinoma from simple hepatic steatosis[18]. New MAFLD diagnosis can emphasize the importance of metabolic disorders in the pathological process from simple benign hepatic steatosis to NASH[19]. According to this simple and comprehensive new diagnostic criteria as well as conceptual frameworks, such as the evaluation of MAFLD-related inflammation and the diagnostic criteria of MAFLD-related hepatocirrhosis, MAFLD diagnostic criteria may improve the level of patient management and even increase the clinical benefits in the future[20].

To understand whether there is a significant difference between the new MAFLD diagnostic criteria and the original NAFLD diagnostic criteria in the prevalence of urban healthy adults in China and to further explore potential differences in examination results between NAFLD patients and MAFLD patients, a cross-sectional study on Asian adults who participate in general health examination was conducted. A total of 795 participants, as a representative sample of a healthy adult population in Shanghai, were recruited by the health examination centre of Wu Song Branch of Zhongshan Hospital, Fudan University in 2020. We also explored whether there was any change in the ability to identify advanced liver fibrosis in the health examination results based on the new MAFLD definition.

## Methods

### Study design

As a cross-sectional study, this survey included a questionnaire survey, structured interviews, physical examinations and laboratory tests in health examination institutions. The ethics committee in Zhongshan Hospital, Fudan University approved this investigation. The health examination centre of Wu Song Branch of Zhongshan Hospital obtained all participants’ written informed consent.

### Study participants and the collection of data

A total of 795 subjects (21–83 years old, average age 45.17) were recruited from the health examination centre of Wu Song Branch of Zhongshan Hospital, Fudan University, including all adults over the age of 18 who participated in the general medical examination in Wu Song Medical Examination Center in the same period. All participants were urban residents of Shanghai. Using a standardized questionnaire survey and physical examination, information on age, height, weight, waist circumference, blood pressure, alcohol consumption, diabetes, hypertension, lipid metabolism disorder and other systemic diseases can be obtained. The participants in this study come from economically developed cities, and they have received a certain degree of education. Therefore, the medical history they provided is reliable. It cannot be ruled out that patients may inadvertently miss the use history of some drugs or dietary supplements that may cause liver damage when collecting information, but we have collected their medical history as much as possible through standardized questionnaires conducted by well-trained doctors. The patient fasted overnight before collecting blood samples. Through the standardized analysis of blood samples and urine samples by the laboratory department in the hospital, the results of fasting blood glucose (FBG), glycosylated hemoglobin (HbA1c), uric acid, creatinine, lipid profile, complete blood count, albumin, alanine aminotransferase (ALT), aspartate aminotransferase (AST), fasting insulin, γ-glutamyl transferase (GGT), C-reactive protein (CRP), high density lipoprotein cholesterol (HDL-C), total cholesterol, triglycerides, low density lipoprotein cholesterol (LDL-C) and some other results can be obtained. All patients underwent abdominal B-ultrasound examination[21, 22]. The diagnosis of hepatic steatosis was made according to the results of abdominal B-ultrasound examination in the imaging department. Four ultrasonographic findings, including hepatorenal echo contrast, liver brightness, deep attenuation, and vessel blurring, were recognized by subjective evaluation of experienced imaging doctors. They were completely masked to individual personal data.

### Diagnosis of NAFLD and MAFLD

NAFLD is an exclusion criterion defined as liver steatosis without other forms of chronic local liver disease or other systemic diseases that may cause liver steatosis, such as viral hepatitis, excessive alcohol consumption, the use of special drugs, endocrine system diseases and other possible reasons[23].

Excessive alcohol intake was defined as > 20 g/day and > 10 g/day for men and women, respectively. Active viral hepatitis was excluded through the detection of hepatitis B virus surface antigen (HBsAg) positive or hepatitis C virus antibody positive. Other conditions leading to hepatic steatosis, such as autoimmune hepatitis, use of special drugs (e.g., amiodarone, methotrexate, tamoxifen, corticosteroids), total parenteral nutrition, inflammatory bowel disease, hypophysis, hypothyroidism and fat atrophy, were excluded through standardized medical history inquiry of staff.

The diagnosis for MAFLD includes the presence of fatty deposition in the liver and at least one of the following 3 factors: overweight measured by excessive body mass index (BMI, > 23 kg/m^2^ in this Asian group), T2DM, or normal weight/wasting together with at least 2 risks of metabolic disorders.

Metabolic disorder was defined as the simultaneous existence of two or more abnormalities of metabolic risk factors: waist circumference ≥ 90 cm in men (≥ 80 cm in women) of this Asian group, blood pressure ≥ 130/85 mmHg or taking antihypertensive drugs for medical treatment, triglyceride ≥ 1.70 mmol/L or using antihyperlipidemic drugs, HDL-C < 40 mg/dL for men (< 50 mg/dL for women) or taking antihyperlipidemic drugs, detection of prediabetes (fasting glucose 5.6–6.9 mmol/L or glycosylated hemoglobin 5.7–6.4%), insulin resistance index evaluated with homeostasis model assessment (HOMA-IR, calculated through fasting insulin/22.5 × fasting blood glucose) ≥2.5, or C-reactive protein (CRP) level > 2 mg/L[1].

To explore the differences between patients with MAFLD and patients identified according to previous NAFLD diagnostic criteria, participants of this study were further divided into four subgroups: healthy people (NAFLD- MAFLD-), patients with NAFLD without overlapping MAFLD (NAFLD + MAFLD-), patients with MAFLD without overlapping NAFLD (NAFLD- MAFLD+), and patients with simultaneous NAFLD and MAFLD (NAFLD + MAFLD+).

### Statistical analysis

A t-test was applied for the comparison of averages between normally distributed data groups, a nonparametric test was used for nonnormally distributed data, and the χ^2^-test was used for the comparison between classified variables. Statistical significance was considered to indicate *P* < 0.05. SPSS 22 software (IBM Corporation) was used for analysis.

## Results

### Patient characteristics

A total of 795 individuals participated in this survey, including 483 men (60.75%) and 312 women (39.25%) at 45.17 ± 10.44 years old. The median BMI was 25.24 kg/m^2^. In addition, 519 participants (65.28%) were overweight (BMI > 23 kg/m^2^), and 396 people (49.81%) had high waistlines. A total of 186 people (23.40%) had a clear history of drinking, of which 25 people had excessive intake of alcohol. A total of 439 people (55.22%) had blood pressure greater than 130/85 mmHg or used antihypertensive drugs, 295 people (37.11%) had insulin resistance (insulin resistance index ≥ 2.5 assessed by steady-state model), 381 (47.92%) had abnormal glucose metabolism associated with prediabetes (assessed by FBG and HbA1c), and 107 (13.46%) had diabetes mellitus. A total of 236 people (29.69%) had triglycerides greater than 1.70 mmol/L, 223 people (28.05%) had low HDL cholesterol, and 135 people (16.98%) had CRP greater than 2 mg/L.

### Prevalence of NAFLD and MAFLD

Among the 795 people participating in this survey, a total of 46.4% (369/795) had abnormal liver ultrasound results for hepatic steatosis. The results of this general population health examination revealed that the prevalence of fatty liver disease was high. Among 369 patients with fatty liver disease, 345 were diagnosed with NAFLD. Twenty-four people were excluded from the NAFLD group, including 11 patients with excessive alcohol consumption, 11 patients with viral hepatitis B (HBsAg positive), 1 patient with active viral hepatitis B and excessive drinking, and 1 patient with viral hepatitis C (hepatitis C antibody-positive).

**Fig. 1:**
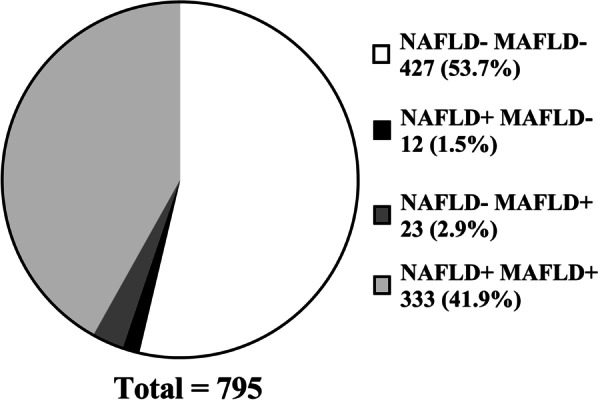
The population and proportion of NAFLD and MAFLD.All participants (n = 795)in this study were divided into four categories according to whether they met the diagnostic criteria of NAFLD or MAFLD. The NAFLD- MAFLD- group had the most people, followed by the NAFLD+ MAFLD+ group. There were fewer members in group NAFLD+ MAFLD- and group NAFLD- MAFLD+.

**Fig. 2:**
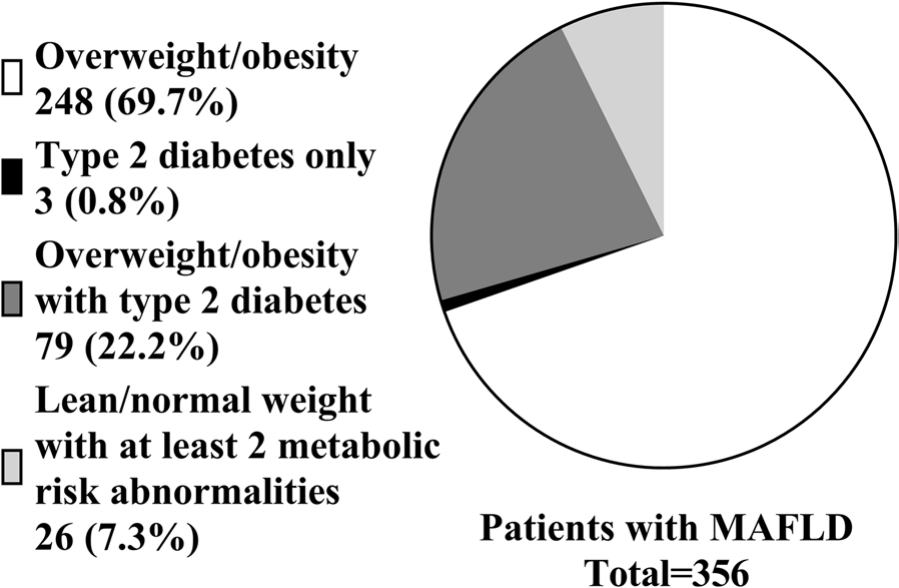
Subgroups of MAFLD patients according to different access criteria.Patients with hepatic steatosis who met the diagnostic criteria of MAFLD (n = 356)were divided into four subgroups according to the three admission criteria of MAFLD: overweight, type 2 diabetes and two or more metabolic risk abnormalities.Patients with MAFLD diagnosed due to overweight/obesity are the most, followed by overweight/obesity patients with type 2 diabetes simultaneously. There were fewer patients who met the diagnostic criteria of MAFLD with at least 2 risks of metabolic disorders or type 2 diabetes as the only abnormality.

According to the definition of MAFLD, 356 of 369 adult hepatic steatosis patients were diagnosed with MAFLD. Among these 356 MAFLD patients, 248 (69.66%) were diagnosed with MAFLD and were classified as exclusively overweight, 3 (0.84%) had normal weight but had type 2 diabetes, 79 (22.19%) were simultaneously overweight with type 2 diabetes, and 26 (7.30%)had normal weight and no diabetes mellitus but had at least two risk factors for metabolic disorders for the diagnosis of MAFLD. (Figs. 1 and 2)

### Differences between NAFLD and MAFLD

Of the 795 people, the prevalence of NAFLD was 43.40% (345/795), and the prevalence of MAFLD was 44.78% (356/795). Among the 795 individuals included in this study, no meaningful difference in the prevalence of NAFLD or MAFLD were noted (Cohen’s Kappa 0.9107, *P* < 0.001, high consistency), and no significant differences in the factors assessed in the examination results, such as age, BMI, waist circumference, FBG, HbA1c, fasting insulin, uric acid, creatinine, lipid profile, complete blood count, albumin, blood pressure, ALT, AST, GGT and other factors, were noted between individuals with NAFLD and those with MAFLD. Differences in the prevalence of overweight, diabetes, hypertension and hyperlipidaemia were noted between the two groups, but these differences were not statistically significant. NAFLD or MAFLD patient groups are highly coincident, and their definitions exhibit strong consistency.

### Differences in characteristics between NAFLD- MAFLD-, NAFLD + MAFLD-, NAFLD- MAFLD + and NAFLD + MAFLD + patients

A total of 427 participants (53.71%) did not suffer from NAFLD or MAFLD (NAFLD- MAFLD-), and 426 of these participants had normal liver ultrasound results. One patient was diagnosed with fatty liver by ultrasound. This patient did not belong to the MAFLD group because no metabolic disorder was found and did not belong to the NAFLD group due to excessive alcohol consumption. Twelve participants (1.51%) had NAFLD but no MAFLD (NAFLD + MAFLD-). Their liver ultrasound findings were abnormal. However, the conditions of overweight/obesity, type 2 diabetes or at least 2 metabolic disorders (MAFLD entry criteria) were not found together with hepatic steatosis in these patients. Twenty-three people (2.89%) had MAFLD but not NAFLD (NAFLD- MAFLD+). They had ultrasound findings of hepatic steatosis and met the admission criteria of MAFLD. However, these participants also had active viral hepatitis or excessive alcohol consumption, meeting the NAFLD exclusion criteria. A total of 333 individuals (41.89%) belonged to both the NAFLD population and MAFLD population (NAFLD + MAFLD+) (Table 1).

**Table 1:** Comparison of measurement results from different patient subgroups

	NAFLD-, MAFLD-	NAFLD + , MAFLD-	NAFLD-, MAFLD +	NAFLD + , MAFLD +	*P* value
Number	53.71% (427/795)	1.51% (12/795)	2.89% (23/795)	41.89% (333/795)	< 0.001
Sex (female/male)	217/210	2/10	5/18	88/245	< 0.001
Overweight (presence/absence)	192/235	0/12	20/3	307/26	< 0.001
Type 2 diabetes mellitus (presence/absence)	25/402	0/12	5/18	77/256	< 0.001
Hypertension (presence/absence)	190/237	1/11	16/7	232/101	< 0.001
Dyslipidemia (presence/absence)	272/155	5/7	20/3	279/54	< 0.001
Age (y)	45 (37, 51)	44 (33.75, 55.5)	53 (40, 57)	46 (39, 53)	0.006
Body weight (Kg)	62 (55, 70)	60 (57, 63)	72 (68, 85) ***##	76 (71, 84) ***###	< 0.001
Body mass index (Kg/m^2^)	23 (21, 25)	22.5 (22, 23)	27 (25, 30) ***###	27 (25, 30) ***###	< 0.001
Waist circumference (cm)	80 (75, 85)	79 (75, 83)	89 (83, 97) ***#	90 (85, 98) ***###	< 0.001
Systolic blood pressure (mmHg)	125 (113, 137)	118.5 (115.25, 126.75)	139 (123, 154) **##	135 (123, 150) ***##	< 0.001
Diastolic blood pressure (mmHg)	74 (67, 82)	70 (65.5, 73.75)	81 (73, 91) **##	82 (74, 90) ***###	< 0.001
Average number of metabolic disorders	1.83 ± 1.36	0.41 ± 0.52	3.34 ± 1.50***###	3.81 ± 1.48***###	< 0.001
Fasting glucose (mg/dL)	5.2 (4.8, 5.5)	4.75 (4.53, 5.23)	5.5 (5, 5.9) ##	5.5 (5.1, 5.9) ***###	< 0.001
HbA1c (%)	5.6 (5.4, 5.9)	5.35 (5.2, 5.6)	5.9 (5.5, 6.2) ##	5.8 (5.6, 6.3) ***###	< 0.001
Fasting insulin (mU/L)	6.78 (4.64, 9.52)	5.89 (5.06, 7.22)	12.74 (8.5, 19.7) ***##	12.1 (8.12, 17.6) ***##	< 0.001
HOMA-IR	1.55 (1.07, 2.22)	1.32 (1.16, 1.68)	2.9 (2.15, 5.31) ***##	2.93 (1.95, 4.52) ***###	< 0.001
Uric acid (mmol/L)	284 (242, 348)	324.5 (283.75, 388)	381 (331, 448) ***	365 (300, 423.5) ***	< 0.001
Creatinine (mmol/L)	65 (53, 76)	72.5 (61.75, 79)	75 (67, 84) **	72 (61, 82) ***	< 0.001
Total cholesterol (mmol/L)	5.11 (4.48, 5.73)	4.64 (4.28, 5.9)	5.1 (4.34, 5.43)	5.33 (4.74, 6.07) ***	< 0.001
LDL cholesterol (mmol/L)	2.94 (2.47, 3.5)	2.74 (2.5, 3.98)	3.01 (2.49, 3.47)	3.36 (2.92, 3.93) ***&	< 0.001
Triglyceride (mmol/L)	0.96 (0.72, 1.33)	0.99 (0.85, 1.31)	1.49 (1.27, 2.23) ***	1.71 (1.2, 2.35) ***##	< 0.001
HDL cholesterol (mmol/L)	1.37 (1.17, 1.57)	1.2 (1.08, 1.37)	1.26 (1.04, 1.44)	1.12 (0.99, 1.31) ***	< 0.001
Albumin (g/L)	45.4 ± 2.57	46.23 ± 2.33	45.6 ± 2.22	45.68 ± 2.46	0.343
Globulin (g/L)	29.2 (27.3, 31.6)	27.55 (26.65, 28.83)	29.3 (27.6, 31.5)	29.4 (27.25, 31.85)	0.382
A/G	1.6 (1.4, 1.7)	1.7 (1.53, 1.78)	1.5 (1.3, 1.7)	1.6 (1.4, 1.7)	0.338
Aspartate aminotransferase (U/L)	20 (17, 25)	22.5 (21, 23.75)	23 (20, 34)	23 (19, 28) ***	< 0.001
Alanine aminotransferase (U/L)	16 (12, 21)	24 (19, 30.5)	24 (17, 44) **	27 (20, 38) ***	< 0.001
γ-glutamyl transpeptidase (U/L)	18 (14, 27)	26 (20.25, 32.75)	47 (31, 75) ***	35 (23, 53) ***	< 0.001
Alkaline phosphatase (U/L)	70 (57, 85)	89.5 (69.25, 98.25)	85 (71, 93)	76 (66, 92) ***	< 0.001
AST/ALT	1.29 (1.05, 1.53)	0.91 (0.7, 1.21)	1 (0.71, 1.15) **	0.86 (0.65, 1.09) ***	< 0.001
Red blood cell count (× 1012/L)	4.95 (4.59, 5.27)	5.22 (4.48, 5.42)	5.29 (4.81, 5.63) *	5.28 (4.93, 5.57) ***	< 0.001
Hemoglobin (g/L)	146 (136, 158)	158.5 (142.5, 161)	163 (148, 168) **	157 (145.5, 165) ***	< 0.001
White blood cell count (× 10^9^/L)	5.5 (4.6, 6.3)	5.5 (5.03, 6.35)	6.2 (5.8, 7) *	6.4 (5.3, 7.4) ***	< 0.001
Percentage of neutrophils (%)	57.85 ± 7.45	54.33 ± 6.33	58.69 ± 7.27	59.12 ± 7.68	0.032
Neutrophil count (× 10^9^/L)	3.14 (2.56, 3.86)	2.93 (2.33, 3.65)	3.75 (3.44, 4.27)	3.69 (3.01, 4.59) ***#	< 0.001
Platelet (× 10^9^/L)	232 (195, 266)	229 (213.5, 243.25)	218 (197, 242)	237 (198, 276.5)	0.272
NFS	-2.05 (-2.92, -1.17)	-2.7 (-3.77, -2.23)	-1.27 (-2.42, -0.67)#	-1.64 (-2.61, -0.88)**	< 0.001
FIB-4	0.95 (0.73, 1.29)	1.04 (0.56, 1.24)	1.07 (0.68, 1.61)	0.87 (0.62, 1.21) *	0.040
NFS (< -1.455)	287/427	10/12	10/23	189/333	
NFS (-1.455 < = NFS < 0.676)	137/427	2/12	13/23	140/333	
NFS (> = 0.676)	3/427	0/12	0/23	4/333	
FIB-4 (< 1.30)	325/427	10/12	15/23	267/333	
FIB-4 (1.30 < = FIB-4 < 1.45)	28/427	1/12	1/23	18/333	
FIB-4 (1.45 < = FIB-4 < 2.67)	71/427	1/12	7/23	45/333	
FIB-4 (2.67 < = FIB-4 < 3.25)	2/427	0/12	0/23	2/333	
FIB-4 (> = 3.25)	1/427	0/12	0/23	1/333	

All data are expressed as the mean ± SD, medians (interquartile range), or n (%), as appropriate. *Difference from NAFLD- MAFLD- group (P<0.05), **P<0.01, ***P<0.001. ^#^Difference from NAFLD+ MAFLD- group (P<0.05), ^##^P<0.01, ^###^P<0.001. &Difference from NAFLD- MAFLD+ group (P<0.05), ^&&^P<0.01, ^&&&^P<0.001.

The levels of ALT, GGT, AST/ALT, uric acid, creatinine, erythrocytes, hemoglobin and leukocytes in MAFLD patients were significantly higher than those in healthy people. Values for BMI, waist circumference, blood pressure, triglyceride, GGT, neutrophil count, fasting insulin and HOMA-IR calculation results were meaningfully higher in patients with MAFLD than in healthy people and NAFLD + MAFLD- patients.

Diastolic blood pressure, glucose and HbA1c were significantly lower in patients from the NAFLD + MAFLD- group than in MAFLD patients. The measured value of the NAFLD + MAFLD- group was even lower than that of the normal population. However, no statistical significance was found. NAFLD + MAFLD- patients seem to have better metabolic conditions because overweight, diabetes and other possible metabolic risk factors were already excluded in this patient population.

In contrast, NAFLD- MAFLD + patients have relatively severe metabolic disorders. The risk of metabolic disorder in these patients was ignored when using the NAFLD diagnostic criteria. The higher proportion of patients in the NAFLD- MAFLD + group cannot rule out the risk of progressive liver fibrosis (NFS with cut-off value − 1.455, χ^2^ 5.115, *P* = 0.0237). No significant differences in glucose metabolism, insulin resistance, liver enzyme level or the number of metabolic disorder risks were noted between NAFLD- MAFLD + patients and NAFLD + MAFLD + patients.

The values for BMI, waist circumference, blood pressure, glucose, HbA1c, fasting insulin and HOMA-IR were significantly increased in patients from the NAFLD- MAFLD + group in comparison with NAFLD + MAFLD- patients. The NFS in NAFLD + MAFLD- patients was significantly lower than that in NAFLD- MAFLD + patients. Due to the restricted number of patients included in these two groups, no other meaningful differences between NAFLD + MAFLD- patients and NAFLD- MAFLD + patients were identified.

Compared with the healthy population, the patient population with simultaneous NAFLD and MAFLD had higher values for fibrosis 4 score (FIB-4), cholesterol, LDL-C, triglycerides, GGT, AST, ALT, NFS leukocyte count and neutrophil count, aspartate aminotransferase-to-platelet ratio index (APRI), whereas HDL-C levels were lower than those noted in the healthy population. Patients with both NAFLD and MAFLD have severe lipid metabolism disorders with high serum levels of liver enzymes, neutrophil counts, NFS and FIB-4. A higher proportion of patients in this group cannot rule out the risk of progressive liver fibrosis (NFS with cut-off value − 1.455, χ^2^ 8.740, *P* = 0.0031).

### MAFLD subgroups based on diagnostic criteria have different clinical features

According to the criteria, after fatty liver is found, patients with MAFLD can be divided into three categories: overweight, T2DM or at least two risk factors for metabolic disorders. Among the patients diagnosed with MAFLD, 8.15% exhibited thin or normal weight, and 91.85% were overweight or obese. Between the two subgroups differentiated by weight, significant differences were noted. In the group of patients diagnosed with MAFLD, the overweight population had higher NFS (*P* = 0.0179), systolic blood pressure (*P* = 0.0369), uric acid (*P* = 0.0155), and HOMA-IR (*P* < 0.0001).

Among patients diagnosed with MAFLD, 23.03% were diabetes patients, whereas 76.97% did not have diabetes. A significant difference was noted between the examination results of the two groups. Diabetes patients had higher ALT (*P* = 0.0111), AST (*P* = 0.0054), age (*P* < 0.0001), systolic blood pressure (*P* = 0.0001), diastolic blood pressure (*P* = 0.0182), body weight (*P* = 0.0133), BMI (*P* = 0.0035) and NFS (*P* < 0.0001).

Among patients diagnosed with MAFLD, 26.40% had drinking habits, and the levels of AST (*P* = 0.0449), GGT (*P* = 0.0006), diastolic blood pressure (*P* = 0.0230), uric acid (*P* = 0.0004) and creatinine (*P* = 0.0001) were significantly higher.

## Discussion

By analyzing the health examination results of 795 participants using the new definition of MAFLD, the prevalence of NAFLD was 43.40% and that of MAFLD was 44.78%. No significant difference was found in the prevalence between NAFLD and MAFLD. Compared with healthy individuals and NAFLD + MAFLD- patients, MAFLD patients had a worse metabolic profile. The MAFLD diagnostic criteria facilitated the identification of metabolic disorders in this subset of patients who had been excluded by the NAFLD criteria due to other concurrent diseases, emphasizing the risk of progressive liver fibrosis in this subset. This finding has important implications for the NAFLD- MAFLD + population, who have relatively severe metabolic disorders.

Fatty liver was present in 46.41% (369/795) of the 795 individuals investigated in this study, and the prevalence of liver steatosis detected by abdominal B-ultrasound was high. Possible reasons were that the health examination centre recruited participants in this study who were mostly from the urban area of Shanghai and mostly city dwellers with relatively affluent family status. In this study, the blood sample examination findings of patients with ultrasound confirmed fatty liver disease were significantly different from those of healthy participants. These findings are highly consistent with previous studies on NAFLD[14, 24].

The harmful effects of hepatic steatosis in the human body are mainly reflected in two aspects: the risk of cardiovascular and cerebrovascular disease due to metabolic disorders and the adverse liver outcomes due to hepatic inflammation and liver fibrosis[18, 25].

In this study, NAFLD- MAFLD + patients had statistically higher blood pressure, BMI, waist circumference, NFS, and abnormal glucose metabolism (e.g., fasting glucose, fasting insulin, glycosylated hemoglobin, insulin resistance index). The NAFLD + MAFLD- and NAFLD- MAFLD + groups differed significantly based on their glucose metabolism profile, with the MAFLD + group exhibiting increased metabolic disorder and a possible trend towards cardiovascular and cerebrovascular disease[8, 26, 27]. However, this study failed to find a significant difference due to the small sample size. The presence of metabolic disorders in this subset of patients is highly overlooked, and their risks of metabolic disorders, steatohepatitis, and advanced liver fibrosis are also difficult to appreciate, representing a blind area for the diagnosis, monitoring, and treatment of NAFLD-related diseases.

As a group of acquired metabolic stress-related disorders, most simple fatty livers are benign, whereas a subset of patients may develop NASH, which is at risk of progression to liver fibrosis and associated complications, including hepatocellular cancer[28]. Therefore, timely detection of NASH and progressive liver fibrosis is very important[29]. Previous studies showed that NAFLD patients with obesity and abnormal glucose metabolism were more likely to have adverse hepatic outcomes, whereas this study found that the NAFLD- MAFLD + group had a higher noninvasive liver fibrosis score NFS than the NAFLD + MAFLD- group. Previous studies have found that the new diagnostic criteria for MAFLD will be more helpful in identifying patients with advanced liver fibrosis than the NAFLD criteria[7, 19]. These results suggest that the MAFLD diagnostic criteria not only alert the population to a greater risk of CVD but also identify a group who may have adverse hepatic outcomes due to fatty liver progression[30, 31]. Other previous studies have shown that NAFLD patients with intercurrent diabetes have different liver disease outcomes than NAFLD patients without diabetes. The prevalence of NASH in NAFLD patients with diabetes was 68–78%, and the rate of progression to fibrosis was 22–60%[32]. The use of MAFLD diagnostic criteria with T2DM and at least 2 risk factors for metabolic disorder or overweight facilitates the identification of patients at high risk for liver disease[33].

There have been studies on MAFLD in the population. A previous study compared the prevalence and incidence of MAFLD and previous NAFLD standards and evaluated the risk of fatty liver patients combined with other risk factors[34]. Another study found a group of NAFLD + MAFLD- patients who will not develop significant liver disease and lack other risk factors for liver injury due to low metabolic burden [35]. A prospective cohort study compared the all-cause mortality of MAFLD patients in different subgroups according to metabolic disorders[36]. However, the noninvasive liver injury evaluation indicators of adverse liver outcomes, such as NASH and advanced liver fibrosis, HOMA-IR calculated using fasting insulin, prediabetes assessed by glycosylated hemoglobin measurements, and the important indicator C-reactive protein (CRP) were not totally available in these studies, making it difficult to accurately reflect the situation in patients. A meta-analysis showed that the new definition of MAFLD is helpful to identify a group of patients who may have serious liver injury[37]. This study verified this conclusion. Most of the studies included in the meta-analysis did not assess fasting insulin, CRP or other examination indicators in patients; thus, the situation of patients was not accurately reflected. On the other hand, the studies included in this meta-analysis came from different regions, and only some of them focused on Asian participants.

This study has some limitations. The population with MAFLD had more severe metabolic abnormalities with a tendency to develop atherosclerosis. However, as the sample size of this group of NAFLD + MAFLD- patients was smaller, the difference only represented a trend, and no further significant results could be found. The relatively small number of samples in this study and the insufficient number of patients with fatty liver make it difficult to detect subtle differences between the two diagnostic criteria. In addition, the population recruited by the health examination centre included only the adult population and urban residents, and the relatively limited representation of this study may not reflect the most realistic and accurate disease prevalence in the whole population. Finally, in the general health examination results of the population investigated in this study, only abdominal B-ultrasound was used to evaluate the patients’ liver lesions. If other techniques are applied, such as proton density fat fraction assessed by magnetic resonance imaging (MRI-PDFF), magnetic resonance spectroscopy (MRS), quantitative ultrasound techniques, biomarker tests for liver fibrosis, and liver biopsy, a more accurate and efficient assessment of liver injury in these patients will be possible in these patients[38, 39]. In the future, further large-scale research on the impact of new MAFLD diagnostic criteria will have important clinical significance.

## Conclusion

In the population participating in this study, the new MAFLD diagnostic criteria have very similar prevalence and patient characteristics compared with previous NAFLD diagnostic criteria but help to identify a group of patients with high risks of metabolic disorders and liver fibrosis who were not identified by NAFLD diagnostic criteria due to excessive drinking or combination with other chronic diseases. In the process of precise treatment and management of high-risk patients in urban adults, compared with the previous NAFLD criteria, MAFLD diagnostic criteria are more inclusive and give more attention to the adverse outcomes that may be caused by risk factors, such as obesity, diabetes and metabolic disorders, which are important influencing factors of liver fibrosis.

## Data Availability

The datasets used and/or analysed during the current study are available from the corresponding author on reasonable request.

## Abbreviations

NAFLD: nonalcoholic fatty liver disease
MAFLD: metabolic dysfunction-associated fatty liver disease
NASH: nonalcoholic steatohepatitis
T2DM: type 2 diabetes
HBsAg: hepatitis B virus surface antigen
ALT: alanine aminotransferase
AST: aspartate aminotransferase
GGT: γ-glutamyl transferase
FBG: fasting blood glucose
HbA1c: glycosylated hemoglobin
HOMA-IR: insulin resistance index evaluated with homeostasis model assessment
LDL-C: low density lipoprotein cholesterol
HDL-C: high density lipoprotein cholesterol
NFS: nonalcoholic liver disease fibrosis score
FIB-4: fibrosis 4 score
APRI: aspartate aminotransferase-to-platelet ratio index
BMI: body mass index
